# Fine Root Traits across Different Root Orders and Their Associations with Leaf Traits in 15 Co-Occurring Plant Species from the Desert–Oasis Transition Zone in the Hexi Corridor, Gansu Province, China

**DOI:** 10.3390/plants13172472

**Published:** 2024-09-04

**Authors:** Yiming Chen, Jing Ma, Hongyong Wang, Tingting Xie, Quangang Li, Lishan Shan

**Affiliations:** College of Forestry, Gansu Agricultural University, Lanzhou 730070, China; chenym@st.gsau.edu.cn (Y.C.); majinggabriel@gmail.com (J.M.); hongyongwang00@gmail.com (H.W.); xieting1026@126.com (T.X.); liquangang2024@126.com (Q.L.)

**Keywords:** root order, economics spectrum, trade-off strategy, desert–oasis transition zone

## Abstract

Fine root traits embody trade-offs between resource acquisition and conservation in plants. Yet, the differentiation of these traits across root orders, the existence of a root economics spectrum (RES) spanning these orders, and their linkage with leaf traits remain underexplored. In this study, we analyzed the first three root orders and leaf traits of 15 co-occurring plant species, including ten herbs and five shrubs, from the desert–oasis transition zone of the Hexi Corridor. We measured twelve morphological and chemical traits to investigate the relationships between root and leaf traits. Our results revealed significant variation in root traits both among species and within species across different root orders. We identified RES that spanned root orders, with higher-order roots exhibiting more conservative traits and lower-order roots displaying traits aligned with resource acquisition. Additionally, leaf and fine root traits showed partially decoupled adaptive strategies, yet evidence also supported the existence of a leaf economics spectrum (LES) and a potentially two-dimensional whole plant economics spectrum (WPES). Our findings suggest synergistic resource allocation strategies between fine roots and the entire plant, emphasizing the importance of root order in understanding fine root structure, function, and their interactions with other plant organs. These insights advance the understanding of fine root traits and their integration within the broader plant economics spectrum. Nevertheless, the differences in fine root traits across root orders, the presence of a root economics spectrum (RES) spanning these orders, and the relationships between fine root and leaf traits remain underexplored. We examined the first three root orders and leaves of 15 co-occurring plant species (ten herbs and five shrubs) from the desert–oasis transition zone in the Hexi Corridor, measured twelve key morphological and chemical traits. We observed substantial variation in root traits among species and root orders within species. The root economics spectrum (RES) spanned across root orders, with higher-order roots positioned at the conservative end and lower-order roots at the acquisitive end of the “investment-return” strategy axis. Leaf and fine root traits of the 15 co-occurring plant species exhibited partially decoupled adaptive strategies. However, there was also evidence for the presence of a leaf economics spectrum (LES) and a whole plant economics spectrum (WPES), with the WPES potentially being two-dimensional. Furthermore, our findings suggest synergistic resource strategies between fine roots and the whole plant. Concurrently, the significant interspecific and intraspecific differences in fine root traits, combined with the presence of a root economics spectrum across root orders, underscore the critical importance of root order in studying fine root structure, function, and their associations with other plant organs. Our findings offer valuable insights for future research on fine root traits, the RES, and their integration with the whole plant economics spectrum.

## 1. Introduction

Plant functional traits are a series of attributes developed through long-term evolution that influence a plant’s ability to colonize, survive, grow, and die in response to environmental adaptation [1,2]. The trade-offs among plant functional traits reflect a balance between resource investment and returns, providing insights into the mechanisms of plant adaptation to their environments [2]. In recent years, the intrinsic linkages and trade-offs between plant traits have garnered significant attention. Notably, Wright et al. [3] introduced the concept of the leaf economics spectrum (LES), a globally applicable framework that describes the continuous variation of leaf traits. This groundbreaking work has expanded the scope of functional trait analysis in plants. Specifically, the leaf economics spectrum delineates the trade-off between resource acquisition and conservatism in vascular plants, with one end embodying a “fast investment-return” strategy and the other a “slow investment-return” strategy [3,4]. Plants adopting the “fast investment-return” strategy typically exhibit higher specific leaf area (SLA), elevated leaf nitrogen concentration (LNC), accelerated photosynthetic rates, and extended lifespans, whereas those following a “slow investment-return” strategy display contrasting traits [5]. Leaves and fine roots are critical resource-acquiring organs both above- and belowground in plants, exhibiting coordinated relationships across various traits [6,7]. However, research on fine roots has lagged behind that of leaves because fine roots directly absorb water and nutrients from the soil [8], making them difficult to sample and certain root traits challenging to measure.

In recent years, research on root systems has increasingly focused on interspecific and intraspecific trait variations [9,10]. On this basis, numerous studies have proposed a root economics spectrum (RES) in conjunction with the hypothesis of trait harmonization between above- and belowground organs [11,12,13]. Two prevailing perspectives dominate discussions on the root economics spectrum (RES). One perspective argues for the existence of a RES similar to the leaf economics spectrum. The higher-order and lower-order roots were placed at the two ends of the RES along the conservation and acquisition strategy axis, respectively [12,14]. An alternative viewpoint posits that RES exhibits multidimensional variations influenced by soil environments and mycorrhizal interactions. However, these studies have predominantly used traditional root diameter classification methods, which obscure the resource acquisition strategies across different root orders.

Traditional definitions classify roots with a diameter ≤ 2 mm as fine roots [15]. While this method facilitates rapid sampling, it fails to capture the structural and functional diversity of fine roots and is unsuitable for cross-species comparisons of roots with similar diameters [16]. Evidence suggests that coarse roots also perform absorptive functions in some woody plants [17], and fine root morphology varies significantly across species [18]. The method of classifying fine root systems into different orders based on river branching patterns addresses the limitations of traditional classification methods [19]. Subsequent research using this method has elucidated structural and functional differences among fine roots of varying orders [20,21,22]. Despite these advances, there is a lack of studies on the resource acquisition strategies of fine roots in the root economics spectrum as classified by root order. 

Previous research has elucidated a coordinated relationship between traits of above- and below-ground plant organs [4,23]. The integration of these functionally similar traits gives rise to the whole plant economics spectrum (WPES). In this context, numerous studies have reported the relationship between analogous traits in above- and below-ground plant organs. For example, Liu et al. [7] discovered that the nitrogen content in the leaves and fine roots of 64 plant species from the semi-arid and arid regions of Northern China co-varied across multiple spatial scales. Similar findings were reported across various scales [6,12,24]. Nevertheless, divergent findings have emerged from several studies, indicating a partial decoupling of leaf and fine root similarity traits [25,26,27]. Consequently, the association between leaf and fine root traits remains inconclusive. Since then, additional studies have found a lack of correlation between LES and RES [28,29]. Most of these studies did not consider root order. Therefore, it is necessary to investigate the relationship between leaf and fine root similar traits and between RES and LES based on previous studies and in conjunction with the root order classification methods.

Given the critical role of fine roots in plant growth and the necessity of research employing the root order classification method, we analyzed 12 morphological and chemical traits of fine roots and leaves from different root orders in 15 co-occurring plant species (ten herbs and five shrubs) located in the desert–oasis transition zone in the Hexi Corrido in order to clarify the strategy of acquiring resources for fine roots from different root sequences and investigated the relationship between the traits of fine roots from different root orders and the traits of leaves as well as the economics spectrum. Based on previous studies, we propose the following Hypotheses: (1) there are significant interspecific and intraspecific (root order) differences in plant root traits; (2) there is a partial decoupling between plant root and leaf traits; (3) a root economics spectrum spans the root order hierarchy, with lower-order roots positioned at the acquisitive end and higher-order roots at the conservative end; and (4) a whole plant economics spectrum exists among co-occurring plant species in the transition zone of the desert oasis, with the RES and WPES being closely linked and highly coordinated.

## 2. Materials and Methods

### 2.1. Study Site and Species Selection

The study area is located near the Linze Inland River Basin Comprehensive Research Station, Chinese Academy of Sciences (100°9′49.6″ E, 39°22′48.4″ N), at the interface between the Badain Jaran Desert and the Linze Oasis. This region features a temperate continental desert steppe climate. The mean annual temperature is 7.6 °C, and the mean annual precipitation is 117 mm, 65% of which is concentrated during the growing season from July to September. The soil is predominantly gray-brown desert soil, noted for its coarse texture and low nutrient content. The landscape of the area is mainly fixed, semi-fixed, and mobile sand dunes, with vegetation coverage of 76%, 56%, and 22%, respectively. The vegetation includes annual herbs (*Bassia dasyphylla*, *Salsola collina* and *Suaeda glauca*) and perennial shrubs (*Nitraria sphaerocarpa*, *Calligonum mongolicum* and *Caragana korshinskii*). We selected 15 plant species from the most common flora in the study area, comprising ten herbs and five shrubs. Details are provided in Table S1.

### 2.2. Sample Collection

**Figure 1:**
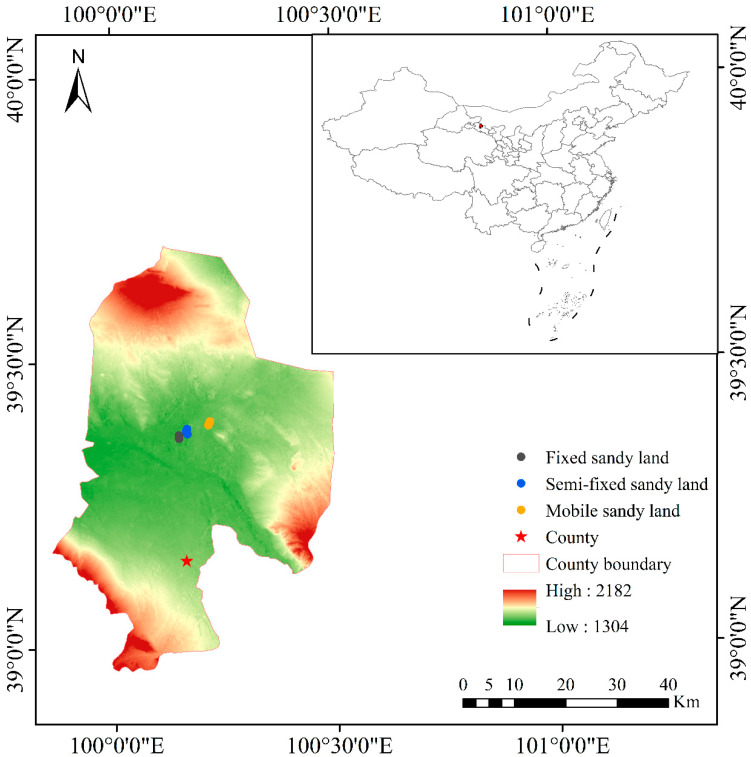
Study area and the distribution of plant quadrat.

In August–September 2022, we collected leaves and fine roots of the 15 selected species within the designated study area. Sample plots were established in ungrazed and ecologically undisturbed reserves, with three plots (20 m × 20 m each) per landscape type, totaling nine plots (Figure 1). To minimize the impact of soil texture differences on plant traits, we selected areas with similar sandy soil distribution when setting up the sample plots. The distance between each landscape type did not exceed 10 km. We collected the selected species from each landscape type. Because the fine root biomass was relatively small, we randomly selected 20 healthy adult plants of each species. When collecting root samples, we dug down from soil blocks located 10 cm away from herbaceous plants and 20 cm away from shrub plants. This distance effectively covered the main distribution area of the roots for both herbaceous and shrub plants while minimizing interference with the roots of neighboring plants. Care was taken to preserve the integrity of root branches during collection, ensuring that the samples represented the full root architecture as much as possible. After the sample collection was completed, we carefully cleaned the soil and impurities from the root system, and then placed the sample into well-marked plastic bags. For each species, we selected 5–15 plants for leaf samples collection. When the same species appeared in multiple landscape types, it was regarded as a different species for leaf collection. All samples were transported in insulated boxes with ice packs to the laboratory for morphological and chemical analysis.

### 2.3. Plant Trait Measurements

The fine root samples were carefully washed with deionized water and classified into different root orders using the Root order classification methods. Briefly, the most distal roots were numbered as first-order roots, and second-order roots began at the junction of two first-order roots [19]. Roots of the same order from the same species and habitat were then homogenized and divided into three equal portions for morphological and chemical trait analysis.

After removing impurities, the fresh weight of leaves and fine roots was measured using an analytical balance. Leaves and roots were scanned with an Epson scanner, ensuring no overlap during scanning. The scanning data were analyzed with WINRHIZO 2008a (Regent Instruments Inc., Quebec City, QC, Canada) software to determine leaf surface area, leaf volume, total root length, root diameter, total root surface area, and root volume. Leaves were then soaked in water overnight to measure their saturated fresh weight. Finally, samples were dried in an oven at 105 °C for 30 min, then at 75 °C for 48 h until reaching constant weight. The dry weights of leaves and roots were then recorded separately. The formulas for trait calculation are as follows:

Leaf dry matter content (LDMC) = Leaf dry weight/Leaf saturated fresh weight(1)

Specific leaf area (SLA) = Leaf area/Leaf dry weight(2)

Leaf tissue density (LTD) = Leaf dry weight/Leaf volume(3)

Degree of fleshiness (DOF) = Leaf fresh weight/Leaf dry weight(4)

Specific root length (SRL) = Total root length/Root dry biomass(5)

Specific root area (SRA) = Total root surface area/Root dry biomass(6)

Root tissue density (RTD) = Root dry weight/Root volume(7)

After measuring the morphological traits, the plant samples were pulverized and sieved through a 0.15 mm mesh for chemical analysis. The organic carbon and total nitrogen concentration was determined using an Elemental Analyzer (VARIO EL III, Elementar). All of measured traits, acronyms, functions and units are provided in Table 1.

### 2.4. Data Analysis

For statistical analyses, each trait for each species was analyzed using the mean value for that species. First, we used a one-way ANOVA to test for variability among fine root traits across root orders and differences in fine root traits across root orders and growth form of plants for each species. A linear mixed model was used to test the effect of growth form and root order on root traits, considering the species as random effects. Second, to assess variation patterns among individual traits and resource economy, we performed principal component analysis (PCA) for fine root traits, leaf traits, and whole plant traits, respectively, (the degree of leaf fleshing was not a LES trait [4,30], so it was not used in the PCA analysis). Then, we used two-way ANOVA to test the effects of growth form and root order on PC1 and PC2, and Pearson correlation analysis to test whether significant correlations existed between plant traits and between traits and the first two components of PCA.

Afterward, the correlation coefficients between traits were used to build a trait network, and we used the Omic Studio tool for visualization of the correlation network (https://www.omicstudio.cn/tool.). Finally, we determined slopes, intercepts and confidence intervals for the regression relationships of fine roots, leaves and whole plants with each other using the standardized major axis (SMA) regression method [31] in the SMATR (V. 2.0) software to analyze the relationships between fine root, leaf and whole plant resource economics. To distinguish whether differences existed between herbs and shrubs, the heterogeneity of the slope was tested. Common slopes were calculated when the slopes were homogeneous, and the existence of common slopes was then analyzed. The statistical analysis and graphing were conducted using SPSS 26.0 and Origin Pro2022 software.

## 3. Results

### 3.1. Differences in Fine Root Traits among Root Orders

**Figure 2:**
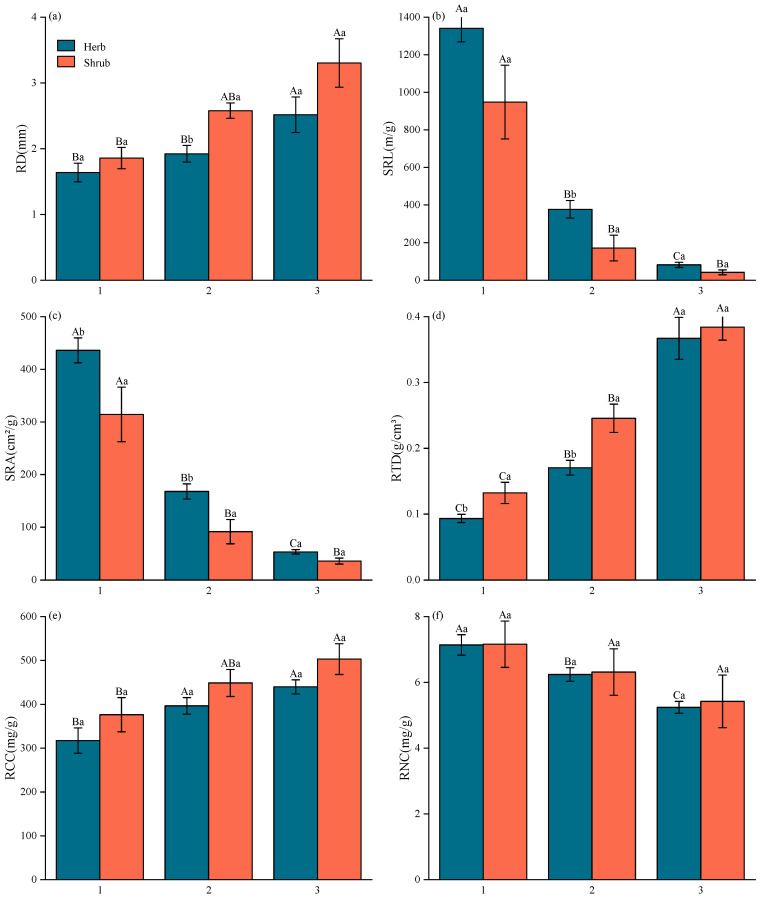
Differences in fine root traits between root orders and between growth forms. (**a**) differences in root diameter (RD) between root orders and between growth forms, (**b**) differences in specific root length (SRL) between root orders and between growth forms, (**c**) differences in specific root area (SRA) between root orders and between growth forms, (**d**) differences in root tissue density (RTD) between root orders and between growth forms, (**e**) differences in root carbon concentration (RCC) between root orders and between growth forms, (**f**) differences in root nitrogen concentration (RNC) between root orders and between growth forms. Data in the figure are mean values ± SE. Different uppercase letters indicate significant differences in plant fine root traits among root orders (*p* < 0.05). Different lowercase letters indicate significant differences in plant fine root traits among growth forms (*p* < 0.05). Abbreviations of traits are provided in Table 1.

In this study, root traits exhibited significant variation among root orders in most species (Table S2). Root morphological traits demonstrated greater variability than chemical traits both among species and among root orders (Table S2, Figure 2). Specifically, root diameter, root tissue density and root carbon concentration increased significantly with root order, while specific root length and specific root area decreased significantly (Table S2, Figure 2a–e). Notably, although the root nitrogen concentration of shrubs did not show a significant difference (*p* > 0.05) across root orders, it still exhibited a declining trend with increasing root order (Figure 2f). Furthermore, linear mixed model analyses assessing the effects of root order and growth form on root traits, with species treated as random effects, revealed that root order significantly influenced all root functional traits. In contrast, growth form significantly affected only RD (F = 7.337, *p* = 0.010), SRL (F = 5.523, *p* = 0.024), SRA (F = 12.582, *p* = 0.001), and RCC (F = 5.232, *p* = 0.028). The interaction between root order and growth form was insignificant for any root traits (Table 2).

### 3.2. Trait Linkages between the Fine Roots of Different Orders and Leaves

**Figure 3:**
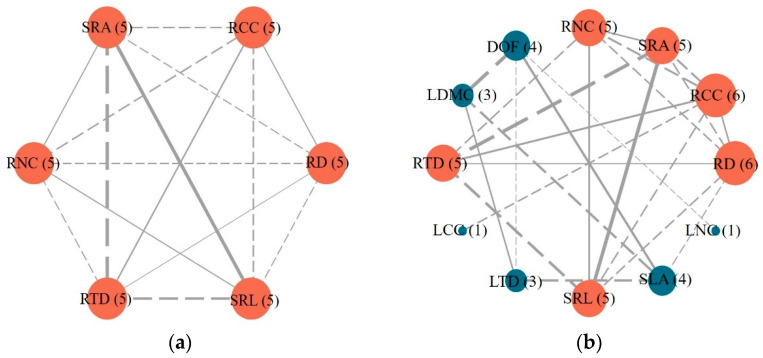
The trait correlation network for fine root (**a**) and whole plant (**b**) traits in 15 plant species. Brown represents fine root functional traits and green represents leaf functional traits. Node size indicates the degree of connectivity. Numbers in parentheses after each trait abbreviation represent the total number of connections between that trait and other traits. The solid line and dashed line represent positive and negative correlations, respectively (*p* < 0.05). Abbreviations of traits are provided in Table 1.

Significant correlations (*p* < 0.05) were found among most functional traits of the 15 co-occurring plant species in the desert–oasis transition zone of the Hexi Corridor. Notably, there was a high degree of connectivity among all six fine root functional traits (Figure 3a, Table S3). In the whole plant traits network, root functional traits had higher connectivity than leaf traits (Figure 3b). Specifically, RCC and RD showed the highest connectivity, followed by RNC, SRA, SRL, and RTD. LCC and LNC demonstrated the lowest connectivity (Figure 3b).

As shown in Table S3, significant negative correlations were observed between SLA and RD (*r* = −0.40, *p* = 0.007) and between LCC and RCC (*r* = −0.44, *p* = 0.003). However, fine root and leaf did not exhibit significant correlations between traits with similar definitions. Specifically, no significant correlations were observed between RNC and LNC (*r* = −0.21, *p* = 0.165), SRL and SLA (*r* = 0.15, *p* = 0.320), or RTD and LTD (*r* = 0.08, *p* = 0.601) (Figure 3a, Table S3).

### 3.3. Leaf, Root, and Whole Plant Economics Spectrum

**Figure 4:**
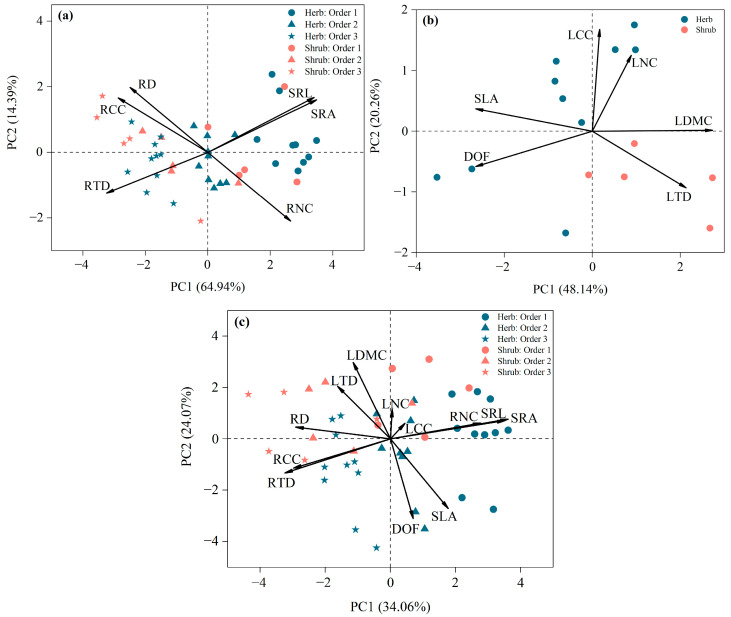
Principal component analysis of fine roots, leaves and whole plant traits: (**a**) shows the principal component analysis of six fine root functional traits; (**b**) shows the principal component analysis of five leaf functional traits; (**c**) shows the principal component analysis of 11 whole plant functional traits. Abbreviations of traits are provided in Table 1.

Variations in plant functional traits on the PCA axis can elucidate resource utilization strategies for fine roots of different root orders, leaves of different growth forms, and whole plants. PCA of fine root traits revealed that the first two dimensions explained 64.94% and 14.39% of the trait variation, respectively, with a cumulative explanation of 79.33% (Figure 4a). All root traits contributed significantly to PC1, with greater contributions than PC2 (Figure 4a, Table 3). As shown in Table 4, PC1 was significantly separated by root order (F = 95.871, *p* < 0.001) and growth form (F = 15.398, *p* < 0.001). Along the PC1 axis, a distinct separation emerges: lower-order roots were characterized by higher SRL, SRA and RNC, alongside lower RD, RTD and RCC. These traits suggest a robust “fast investment-return” strategy, indicative of a resource acquisitive strategy (Figure 2 and Figure 4a). Conversely, higher-order roots, with reduced SRL, SRA, and RNC but increased RD, RTD, and RCC, exemplify a “slow investment-return” strategy, emblematic of a resource-conservative strategy (Figure 2 and Figure 4a).

PCA of leaf traits revealed that the first two dimensions accounted for 44.86% and 22.65% of the trait variation, respectively, with a cumulative total of 67.51% (Figure 4b). Morphological traits contributed predominantly to PC1. In contrast, chemical traits were more significant contributors to PC2 (Table 3). Herbs were primarily positioned on the resource acquisition side of the PCA, whereas shrubs were clustered on the resource conservation side (Figure 4b).

PCA of whole plant traits revealed that the first two dimensions explained 37.00% and 20.10% of the trait variance, respectively, with a cumulative total of 57.10% (Figure 4c). The PC1 axis was primarily influenced by root traits, whereas the PC2 axis was mainly influenced by leaf traits (Table 3). Analysis of variance (ANOVA) of PCA scores for leaf and root traits indicated that both PC1 and PC2 were significantly affected by growth form (F = 36.552, *p* < 0.001; F = 18.473, *p* < 0.001) (Table 4). Herbs were positioned on the acquisition side, exhibiting a “fast investment-return” strategy, while shrubs were clustered on the conservation side, demonstrating a “slow investment-return” strategy (Figure 4c).

### 3.4. Linkages among Roots, Leaves, and Whole Plant Economics Spectrum of 15 Plant Species

**Figure 5:**
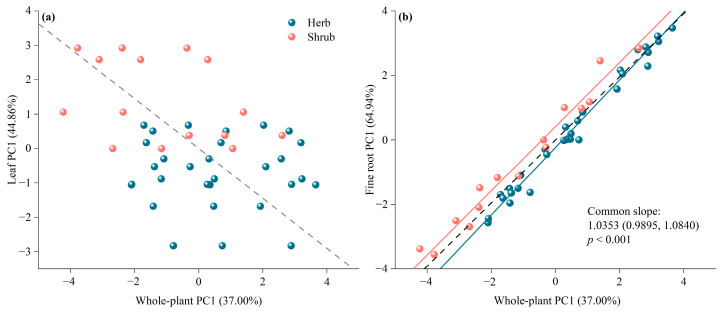
Regression relationships among fine roots, leaves and whole plant PC1: (**a**) shows the relationship between leaf PC1 and whole plant PC1; (**b**) shows the relationship between fine root PC1 and whole plant PC1. Green dots represent herbs, red dots and lines represent shrubs, and black dashed lines indicate no distinction between growth forms. Regression lines are not drawn when the regression relationship is *p* > 0.05. Abbreviations of traits are provided in Table 1.

Bivariate correlations between fine roots, leaves, and whole plant PC1 were assessed using the standardized major axis regression method. Without distinguishing between growth forms, there was a significant negative correlation between leaf PC1 and whole plant PC1 (R^2^ = 0.146, *p* = 0.01) (Figure 5a), and a significant positive correlation between fine root PC1 and whole plant PC1 (R^2^ = 0.958, *p* < 0.001) (Figure 5b). When distinguishing between growth forms, no significant correlation was found between leaf PC1 and whole plant PC1 for both herbs and shrubs (*p* > 0.05). However, a significant positive correlation was observed between fine root PC1 and whole plant PC1 for both herbs (R^2^ = 0.983, *p* < 0.001) and shrubs (R^2^ = 0.971, *p* < 0.001) (Figure 5). There was no significant difference (*p* > 0.05) in the slopes of the relationship between fine root PC1 and whole plant PC1 for herbs and shrubs, indicating a common slope between them. However, a significant difference (*p* < 0.05) was found in the intercepts, with the intercept for herbs being significantly larger than that for shrubs (Figure 5b). Additionally, there was no significant correlation (*p* > 0.05) between leaf PC1 and fine root PC1, regardless of whether growth forms were distinguished (Figure 5).

## 4. Discussion

### 4.1. Differences and Associations between Fine Root Traits and Leaf Traits in Different Root Orders

The root is the primary organ through which plants directly obtain water and nutrients from the external environment. The interspecific and intraspecific variation in root functional traits reflects trade-offs between resource acquisition and storage strategies across different species and highlights the plasticity of individual responses to environmental changes [32,33]. Studies investigating fine root traits among both symbiotic plants in semiarid inland dunes and subtropical woody species have consistently revealed substantial disparities in fine root functional traits at both interspecific and intraspecific (root order) levels [5,10]. These findings align closely with our observations (Table S2, Figure 2), strongly supporting our Hypothesis (1). Specific root length, a critical trait defining the capacity of roots to uptake water and nutrients while quantifying root turnover dynamics, emerges as a comprehensive indicator of root physiology and its adaptive plasticity in response to environmental [11,34]. We found significant differences in SRL between and within species, suggesting a diverse array of soil resource acquisition strategies among different species and root orders [9]. Additionally, our results showed that the variation in morphological traits of fine roots was significantly greater than that of chemical traits (Table S2, Figure 2). This finding is consistent with the work of Comas and Eissenstat [9] and Chen [35], suggesting that morphological and structural characteristics of roots can change significantly across species or in different environments. In contrast, the concentration of biologically essential elements, such as nitrogen, remains relatively stable. This may be related to root evolution and the characterization of crucial root functions [36].

We observed that root orders had a significantly greater impact on root functional traits than growth forms, corroborating findings by Yu et al. [5]. Thus, future studies on root functional traits should prioritize the role of root order. In previous studies on root traits, the traditional classification method was mostly used to identify roots with a diameter ≤ 2 mm as fine roots, which was not conducive to the comparison of fine root morphology and physiology among different species [37]. In contrast, categorizing fine roots by root order offers a more precise characterization of their physiological processes [16].

As the primary nutrient organs above- and belowground, leaves, and fine roots have developed corresponding trait combinations through long-term environmental adaptations, reflecting trait trade-offs [38]. In the desert–oasis transition zone, we observed a significant negative correlation between SLA and RD (*r* = −0.40, *p* = 0.007). This correlation likely arises because SLA reflects a plant’s resource acquisition capacity [3], with larger SLA indicating higher photosynthetic efficiency. Fine roots primarily absorb nutrients and water [39], and smaller root diameters enhance resource acquisition [10]. Therefore, plants have evolved finer roots to ensure an adequate supply of photosynthetic substrates. The significant negative correlation between LCC and RCC (*r* = −0.44, *p* = 0.003) may be attributed to the fact that plants are able to adapt to resource-limited environments by optimizing the allocation of resources through the adjustment of different functional traits [40,41,42]. In addition, the correlation between RNC and LNC in our study was not significant (*r* = −0.21, *p* = 0.165), which is inconsistent with the results of studies of leaf and root nutrient concentrations at different spatial scales [27,43]. This may be due to the fact that in arid habitats with intense light, plants tend to invest more nitrogen concentration in their leaves [26]. This nitrogen is not only involved in photosynthesis but also serves as storage and defense as well as coping with higher solar radiation [44]. In contrast, roots do not seem to need higher nitrogen concentration to cope with these environmental stress challenges due to their deep underground burrowing. Specific root length and specific leaf area are critical traits within the whole-plant economics spectrum, representing the resource acquisition capacities of roots and leaves. Resource economics spectrum theory suggests these traits should function similarly and be highly coordinated [3,4]. However, we found no significant correlation between SRL and SLA (*r* = 0.15, *p* = 0.320), consistent with findings from previous empirical studies [35,38]. Previous studies have reported positive [5,7,45] and negative [46,47] correlations between specific root length and specific leaf area. The variation in these findings may stem from several factors: root system function is influenced by branching order and mycorrhizal colonization, and the phylogenetic conservatism of root traits is stronger than that of leaves [26]. Additionally, the SRL–SLA relationship may vary with spatial scales [45]. Therefore, SRL may not be a functionally similar trait to its SLA counterpart in arid desert habitats [26]. Future studies should focus on the functions of fine roots across different root orders, plant–mycorrhizal interactions, and the effects of environmental gradients.

Overall, researchers have extensively studied the relationships between leaf and root traits. Some studies have found coordination between corresponding traits (e.g., SLA vs. SRL, LNC vs. RNC) [25,48,49], while others have reported decoupled evolution of these traits [29,50,51]. Our findings support the hypothesis of partial decoupling between leaf and root traits, aligning with our Hypothesis (2). This decoupling suggests that leaf and root traits can vary independently, enabling species with different above- and below-ground phenotypes to coexist locally [17,52]. Despite these findings, there is no consensus, and the factors driving this decoupling remain unexplored. Future research should investigate the corresponding traits of leaves and roots across different habitats and species to elucidate the mechanisms behind this phenomenon.

### 4.2. Characterization of the Economics Spectrum of 15 Plant Resources

Our study revealed significant correlations among the functional traits of fine roots across different root orders of 15 co-occurring plant species in the desert–oasis transition zone of the Hexi Corridor. The intrinsic relationships among fine root traits, along with the multi-trait variations on the PCA axis, collectively determined the trade-offs in resource allocation to the underground parts of plants in this region (Figure 3a and Figure 4a, Table 3). Thus, the correlations between functional traits of fine roots in different root orders confirmed the existence of a root economics spectrum across root orders, which is consistent with our hypothesis. In the root economics spectrum, lower-order roots were located at the resource acquisition end of the “investment-return” strategy axis, while higher-order roots were located at the resource conservation end. This finding is consistent with the results of Yu et al. [5] and Li et al. [10]. Specifically, traits representing resource acquisition strategies (SRL, SRA, RNC) gradually decreased, while traits representing resource conservation strategies (RD, RTD, RCC) gradually increased with increasing root order [20,22]. Fine roots are responsible for water and nutrient uptake, with their anatomical features influencing nutrient uptake capacity. Differences in morphological traits among root orders further characterize this capacity [53,54]. We observed a significant increase in RD and RTD, alongside a significant decrease in SRL and SRA, with increasing root order. These findings suggest that higher root orders require greater dry matter investment to enhance root tissue density [45]. In terms of chemical traits, we found that RCC was significantly and negatively correlated with RNC (*r* = −0.59, *p* < 0.001). As root order increased, RCC was larger, RNC was smaller, construction costs were higher, root activity was lower, and maintenance costs were lower [55]. 

We found that the leaf functional traits of plants in the desert–oasis transition zone partially adhered to the leaf economics spectrum. Specifically, the specific leaf area exhibited a significant negative correlation between leaf dry matter content and leaf tissue density. This indicates that plants in this region adapt to harsh environmental conditions by modifying their leaf morphology and structure. Such adaptations likely reduce water loss and enhance photosynthetic efficiency, thereby maintaining plant growth and nutrient cycling [56]. These findings support the applicability of the LES in desert–oasis transition zones. However, contrary to the hypotheses of the leaf economics spectrum theory [4,30], the correlation between leaf nitrogen concentration and both specific leaf area and leaf dry matter content was not significant. This finding suggests that LNC may not be directly linked to resource acquisition strategies in nutrient-poor habitats [26,57]. First, arid plants in northwestern China exhibit substantial nitrogen accumulation [58]. These accumulated nitrogen reserves not only participate in photosynthesis but also serve storage and defensive functions, as well as help cope with increased solar radiation [44]. Second, most of the 15 plant species we collected had highly succulent leaves, which typically exhibit smaller leaf areas and higher water content. These characteristics maximize water storage, reflect sunlight, and reduce transpiration, thereby enhancing the plant’s ability to cope with arid environments, albeit at the expense of some photosynthetic efficiency [51]. We also found leaf morphological traits predominantly loaded on the PC1 axis, whereas chemical traits primarily loaded on the PC2 axis. These findings suggest that co-occurring plants in the desert–oasis transition zone employ flexible strategies to adapt to arid, water-scarce conditions. By optimizing water-use efficiency and photosynthetic capacity, these plants aim to survive at minimal cost [59,60].

For whole plants, fine root traits predominantly loaded on PC1 and were significantly correlated with PC1, while leaf traits primarily loaded on PC2, with most leaf traits also significantly correlated with PC2 (Figure 4c, Table 3). These findings confirm the presence of the whole plant economics spectrum. Previous studies have similarly identified multiple dimensions of trait variation in forest and coastal dune ecosystems [24,28,61]. We found that a single dimension explained only 37.00% of the overall trait variation, suggesting that variation in plant traits in the transition zone of the desert–oasis does not follow a single “fast-slow” strategy [61]. The first dimension represents a trade-off in fine root strategies, with lower-order roots associated with resource acquisition at one end and higher-order roots associated with resource conservation at the other. The second dimension reflects partial leaf economic strategies, with herbs exemplifying a rapid resource acquisition strategy characterized by thin leaves and low tissue density and shrubs exemplifying a resource conservation strategy with the opposite leaf traits. Additionally, we observed distinct trait combinations in herbs and shrubs. Herbs followed a resource acquisition strategy with high SRL, SRA, and SLA. Conversely, shrubs adhered to a conservative strategy with high RD, LTD, and LDMC. These findings suggest that leaf and fine root traits related to the acquisition and utilization of light, water, and nutrients differ between these growth forms [62]. Based on these observations, we predict that a whole plant economics spectrum of at least two dimensions may exist for plants in the desert–oasis transition zone.

Our study revealed a significant positive correlation between fine root PC1 and whole plant PC1 in herbs and shrubs. This significant positive correlation was also observed when growth forms were not distinguished. These findings indicate a close and highly coordinated relationship between RES and WPES, consistent with our Hypothesis (4). The presence of a common slope between the fine root PC1 and whole plant PC1 relationships among herbs and shrubs within the desert–oasis transition zone suggests that these species employ similar resource strategies at both fine roots and whole plant levels [4,11]. This phenomenon may stem from environmental filtering, which suppresses or eliminates species with extreme traits. Over time, this process reduces the distribution range of functional traits within the community, ultimately resulting in a convergence of plant functional traits [35,63,64]. However, significant differences were observed in the intercepts of the relationships between fine root PC1 and whole plant PC1 in herbs and shrubs. This indicates that different growth forms exhibit distinct approaches to constructing WPES, which is reflected in herb and shrub phenotypes and resource strategies [65,66,67]. In addition, we found no significant correlation between PC1 of herb and shrub leaves and PC1 of fine roots, further supporting the notion that leaf and root traits can vary independently as an adaptation to different environments [17,52]. Generally, the relationship between fine roots and whole plants in co-occurring plant species from the desert–oasis transition zone is closer, indicating a tendency for plants in this region to rely on fine roots to sustain normal life activities [7].

## 5. Conclusions

In conclusion, by analyzing the fine root and leaf traits of 15 co-occurring plant species from the desert–oasis transition zone in the Hexi Corridor, we revealed differences in fine root traits across root orders and their associations with leaf traits. First, we observed significant differences in fine root traits between and within species (root orders), with root orders exerting a substantially greater influence on fine root traits than growth forms. Therefore, future studies on fine root functional traits should account for root orders. Second, we observed a decoupling between fine root traits and certain leaf traits, indicating that above- and below-ground traits can vary independently in plants from this region. Third, our study confirmed the existence of the root economics spectrum across root orders. Higher-order roots were positioned at the resource-conservative end of the “investment-return” strategy axis, while lower-order roots were positioned at the resource acquisition end. Additionally, there is evidence to suggest that the whole plant economics spectrum exists in a multidimensional form. Finally, we also found a resource strategy of synergistic changes between plant fine roots and whole plants in the region. Collectively, the 15 co-occurring plant species from the desert–oasis transition zone in the Hexi Corridor exhibit distinct evolutionary and ecological strategies. These species may prioritize the growth of their subterranean parts, favoring the development of fine roots to efficiently access underground resources, thereby enhancing their survival in harsh environments. To advance our understanding of plant root function and the whole plant economics spectrum, future research should focus on the structure and function of roots across different mycorrhizal types, species, and habitats. Additionally, the role of organs beyond leaves and roots in shaping WPES warrants further investigation.

## Figures and Tables

**Table 1:** Measured trait indicators and abbreviations.

Organ	Functional Trait	Functions	Acronym	Unit
Leaf	Leaf dry matter content	Defense	LDMC	mg/g
	Specific leaf area	Resource capture	SLA	cm^2^/g
	Leaf tissue density	Defense	LTD	g/cm^3^
	Degree of fleshiness	Defense	DOF	/
	Leaf carbon concentration	Defense	LCC	mg/g
	Leaf nitrogen concentration	Resource capture	LNC	mg/g
Fine root	Root diameter	Transport, construction, defense	RD	mm
	Specific root length	Resource capture	SRL	cm/g
	Specific root area	Resource capture	SRA	cm^2^/g
	Root tissue density	Transport, construction, defense	RTD	g/cm^3^
	Root carbon concentration	Transport, construction, defense	RCC	mg/g
	Root nitrogen concentration	Resource capture	RNC	mg/g

**Table 2:** Results of linear mixed modeling of the effects of root order and growth form on the fine root traits, considering species as the random effect. Abbreviations of traits are provided in Table 1.

Root Trait	Root Order (RO)		Growth Form (GF)		RO × GF	
	F	*p* Value	F	*p* Value	F	*p* Value
RD	**10.864**	**<0.001**	**7.337**	**0.010**	0.710	0.498
SRL	**34.548**	**<0.001**	**5.523**	**0.024**	1.777	0.183
SRA	**95.946**	**<0.001**	**12.582**	**0.001**	2.219	0.122
RTD	**41.99**	**<0.001**	2.400	0.129	1.029	0.367
RCC	**8.128**	**0.001**	**5.232**	**0.028**	0.016	0.984
RNC	**7.307**	**0.002**	0.056	0.814	0.014	0.986

The bold values denote significant terms of root order and growth form and their interaction on the fine root traits of fine roots at *p* < 0.05.

**Table 3:** Correlation coefficients between root, leaf and whole plant traits and the first and second axes of PCA (PC1, PC2) for 15 plant species. Abbreviations of traits are provided in Table 1.

Traits	Fine Root		Leaf		Whole-Plant	
	PC1	PC2	PC1	PC2	PC1	PC2
RD	−0.659 **	0.434 **	/	/	−0.698 **	0.183
SRL	0.899 **	0.367 *	/	/	0.867 **	0.198
SRA	0.920 **	0.351 *	/	/	0.893 **	0.196
RTD	−0.858 **	−0.272	/	/	−0.815 **	−0.339 *
RCC	−0.760 **	0.364 *	/	/	−0.748 **	−0.237
RNC	0.702 **	−0.460 **	/	/	0.690 **	0.066
LDMC	/	/	0.840 **	0.097	−0.215	0.799 **
SLA	/	/	−0.889 **	0.019	0.377 *	−0.815 **
LTD	/	/	0.833 **	−0.205	−0.360 *	0.728 **
LCC	/	/	−0.058	0.785 **	0.123	0.094
LNC	/	/	0.224	0.681 **	0.038	0.290

Significant correlations are indicated by ‘*’; ‘*’ for *p* < 0.05, ‘**’ for *p* < 0.01.

**Table 4:** Response of 15 plant root, leaf and whole plant traits scored on the first and second axes of PCA (PC1, PC2) to root order and growth form. Abbreviations of traits are provided in Table 1.

		Growth Form (GF)		Root Order (RO)		GF × RO	
		F	*p*	F	*p*	F	*p*
Root traits	PC1	**15.398**	**<0.001**	**95.871**	**<0.001**	0.341	0.713
	PC2	0.162	0.689	0.867	0.428	0.666	0.519
Leaf traits	PC1	**11.146**	**0.005**	/	/	/	/
	PC2	3.209	0.097	/	/	/	/
Whole-plant traits	PC1	**36.552**	**<0.001**	**77.035**	**<0.001**	0.248	0.782
	PC2	**18.473**	**<0.001**	2.604	0.087	0.049	0.952

The bold values denote significant terms at *p* < 0.05.

## Data Availability

Data are contained within the article.

## Supplementary Materials

The following supporting information can be downloaded at: https://www.mdpi.com/article/10.3390/plants13172472/s1, Table S1: Species survey of experimental sites. The table shows the total number of species in each test site; Table S2: Root traits of 12 species among different root orders. The data in the table are mean values ± SE, and the same lowercase letters indicate that there is no significant difference between the root orders at *p* < 0.05. The abbreviations of species and traits are the same as Table 1 and Table S1; Table S3: Pearson correlation coefficient matrix among root and leaf traits of 15 plant species. The lower and upper triangles represent the correlation coefficient and *p* values, respectively. The values of the bold text indicate a significant correlation at *p* < 0.05.
