# Supplementary material for: Fine Root Traits across Different Root Orders and Their Associations with Leaf Traits in 15 Co-Occurring Plant Species from the Desert–Oasis Transition Zone in the Hexi Corridor, Gansu Province, China
Source: Plants (Basel). 2024 Sep 4;13(17):2472. doi: 10.3390/plants13172472 (PMC11396981; doi:10.3390/plants13172472)
Supplement: Supplementary file 1 [file plants-13-02472-s001.zip › plants-3079217-supplementary.pdf]

Table S1. Species survey of experimental sites. The table shows the total number of species in each test site.

Landscape type	Species	Family	Life forms	Number of plants (individuals)	Abbreviation
Fixed dunes	*Karelinia caspia*	Asteraceae	Herb	213	*KC*
	Echinops gmelinii	Asteraceae	Herb	56	*EG*
	Artemisia desertorum	Asteraceae	Herb	46	*AD*
	Inula salsoloides	Asteraceae	Herb	32	*IS*
	Salsola collina	Amaranthaceae	Herb	60	*SC*
	Suaeda glauca	Amaranthaceae	Herb	29	*SG*
	Bassia dasyphylla	Amaranthaceae	Herb	56	*GD*
	Halogeton arachnoideus	Amaranthaceae	Herb	31	*HA*
	Cynanchum chinense	Apocynaceae	Herb	50	*CC*
	Haloxylon ammodendron	Amaranthaceae	Shrub	25	*HAM*
	Calligonum mongolicum	Polygonaceae	Shrub	21	*CM*
	Reaumuria songarica	Tamaricaceae	Shrub	115	*RS*
	Nitraria sphaerocarpa	Nitrariaceae	Shrub	38	*NS*
	Caragana korshinskii	Fabaceae	Shrub	22	*CK*
Semi-fixed dunes	*Bassia dasyphylla*	Amaranthaceae	Herb	1710	*GD*
	Corispermum squarrosum	Amaranthaceae	Herb	276	*CS*
	Haloxylon ammodendron	Amaranthaceae	Shrub	33	*HAM*
	Calligonum mongolicum	Polygonaceae	Shrub	32	*CM*
	Nitraria sphaerocarpa	Nitrariaceae	Shrub	61	*NS*
Mobile dunes	*Corispermum squarrosum*	Amaranthaceae	Herb	681	*CS*
	Artemisia desertorum	Asteraceae	Herb	180	*AD*
	Nitraria sphaerocarpa	Nitrariaceae	Shrub	35	*NS*

Table S2. Root traits of 12 species among different root orders. The data in the table are mean values ± SE, and the same lowercase letters indicate that there is no significant difference between the root orders at $p < 0.05$. The abbreviations of species and traits are the same as Table 1 and Table S1.

Growth form	Species	Root order	RD (mm)	SRL (cm/g)	SRA (cm²/g)	RTD (g/cm³)	RCC (mg/g)	RNC (mg/g)
Herbs	*KC*	1	1.53 ± 0.20b	1205.43 ± 381.75a	429.54 ± 86.85a	0.08 ± 0.01a	221.73 ± 42.40b	6.15 ± 0.25a
		2	2.54 ± 0.10ab	383.13 ± 220.67ab	195.79 ± 69.54b	0.12 ± 0.01a	407.44 ± 30.11a	5.82 ± 0.23a
		3	4.01 ± 0.99a	41.45 ± 13.11b	45.82 ± 13.68b	0.31 ± 0.11a	448.70 ± 42.73a	4.68 ± 0.16b
	EG	1	1.33 ± 0.30c	1306.77 ± 64.06a	413.93 ± 28.71a	0.10 ± 0.01c	241.56 ± 48.85a	7.50 ± 0.18a
		2	2.10 ± 0.04b	159.69 ± 10.89b	105.62 ± 8.80b	0.18 ± 0.02b	309.76 ± 55.07a	6.95 ± 0.52a
		3	3.04 ± 0.15a	49.63 ± 2.94b	47.28 ± 3.21b	0.28 ± 0.03a	361.37 ± 44.13a	5.01 ± 0.26b
	AD	1	1.56 ± 0.16b	968.56 ± 68.34a	332.63 ± 32.04a	0.12 ± 0.02b	225.07 ± 86.80b	6.47 ± 0.37a
		2	2.00 ± 0.23b	198.32 ± 23.49b	116.58 ± 5.04b	0.19 ± 0.02ab	400.06 ± 14.56a	6.12 ± 0.46a
		3	3.31 ± 0.42a	53.50 ± 3.85c	54.35 ± 5.52c	0.27 ± 0.06a	434.94 ± 24.87a	5.61 ± 0.43a
	IS	1	1.14 ± 0.08b	1110.82 ± 131.88a	391.62 ± 17.65a	0.09 ± 0.00b	273.30 ± 18.40a	7.81 ± 0.24a
		2	2.48 ± 0.26ab	716.23 ± 308.25ab	274.50 ± 90.07a	0.11 ± 0.01b	338.57 ± 77.87a	6.04 ± 0.21b
		3	3.45 ± 0.73a	69.44 ± 25.76b	61.30 ± 8.03b	0.22 ± 0.04a	397.52 ± 61.28a	5.00 ± 0.32c
	SC	1	1.28 ± 0.26b	1413.41 ± 102.05a	437.10 ± 40.94a	0.10 ± 0.01c	219.17 ± 31.97b	7.08 ± 0.53a
		2	1.34 ± 0.04b	380.77 ± 38.33b	159.88 ± 12.39b	0.19 ± 0.01b	314.10 ± 49.26ab	6.07 ± 0.32ab
		3	2.82 ± 0.26a	48.24 ± 6.70c	41.79 ± 2.95c	0.35 ± 0.03a	406.13 ± 4.66a	5.09 ± 0.29b
	SG	1	2.36 ± 0.28a	1520.13 ± 178.89a	418.51 ± 37.12a	0.11 ± 0.01c	347.04 ± 40.65a	9.30 ± 0.87a
		2	1.78 ± 0.37a	357.55 ± 42.04b	152.85 ± 19.26b	0.20 ± 0.03b	398.23 ± 33.91a	7.67 ± 0.49ab
		3	1.65 ± 0.87a	111.13 ± 13.59b	57.12 ± 5.13c	0.43 ± 0.03a	409.27 ± 51.76a	6.72 ± 0.52b
	GD	1	2.19 ± 0.11a	1638.78 ± 158.26a	524.68 ± 41.38a	0.08 ± 0.01a	462.27 ± 48.78a	6.92 ± 0.41a
		2	1.88 ± 0.33ab	506.49 ± 20.34b	183.55 ± 10.13b	0.19 ± 0.01b	456.28 ± 46.10a	6.33 ± 0.46ab
		3	1.48 ± 0.04b	147.70 ± 16.40c	68.00 ± 6.19c	0.42 ± 0.04c	491.25 ± 39.84a	5.24 ± 0.28b
	HA	1	1.56 ± 0.53a	1081.02 ± 176.54a	326.59 ± 32.76a	0.13 ± 0.01c	368.64 ± 5.84a	6.23 ± 0.23a

		2	1.35 ± 0.03a	308.47 ± 18.18b	130.40 ± 5.43b	0.23 ± 0.01b	381.70 ± 16.34a	6.03 ± 0.30a
		3	1.86 ± 0.10a	70.96 ± 6.63b	41.36 ± 3.45b	0.53 ± 0.05a	411.98 ± 17.44a	5.34 ± 0.56a
	CC	1	1.14 ± 0.01c	1515.08 ± 74.60a	541.48 ± 29.56a	0.07 ± 0.00c	346.11 ± 9.7b	7.99 ± 0.35a
		2	1.59 ± 0.03b	339.72 ± 29.16b	169.39 ± 11.16b	0.15 ± 0.01b	474.88 ± 27.9a	6.30 ± 0.44b
		3	2.13 ± 0.21a	55.53 ± 7.55c	36.16 ± 1.89c	0.53 ± 0.03a	515.96 ± 48.15a	5.18 ± 0.59b
	CS	1	2.29 ± 0.31a	1645.19 ± 151.75a	543.78 ± 34.08a	0.07 ± 0.00c	467.32 ± 70.74a	5.92 ± 0.29a
		2	2.15 ± 0.33a	425.43 ± 15.22b	190.70 ± 8.68b	0.15 ± 0.01b	483.60 ± 69.02a	5.07 ± 0.22ab
		3	1.51 ± 0.03a	175.05 ± 26.33b	82.09 ± 10.96c	0.35 ± 0.04a	519.73 ± 56.46a	4.53 ± 0.34b
Shrubs	*HAM*	1	1.87 ± 0.04b	1777.45 ± 59.87a	521.38 ± 15.25a	0.08 ± 0.00c	359.20 ± 15.09b	5.05 ± 0.31a
		2	2.36 ± 0.23b	117.93 ± 24.38b	79.30 ± 9.44b	0.23 ± 0.01b	467.59 ± 44.83ab	4.38 ± 0.49a
		3	4.51 ± 0.31a	20.13 ± 3.12b	27.13 ± 2.67c	0.34 ± 0.02a	522.28 ± 49.22a	3.79 ± 0.39a
	CM	1	2.21 ± 0.13b	713.67 ± 143.16a	234.81 ± 38.73a	0.17 ± 0.02c	517.04 ± 57.65a	6.35 ± 0.33a
		2	3.09 ± 0.33a	92.75 ± 33.12b	63.42 ± 15.55b	0.28 ± 0.03b	559.64 ± 38.85a	5.62 ± 0.28a
		3	3.41 ± 0.08a	25.32 ± 1.85b	27.09 ± 2.05b	0.45 ± 0.04a	617.20 ± 47.29a	4.22 ± 0.14b
	RS	1	1.54 ± 0.29b	571.95 ± 48.23a	212.17 ± 8.43a	0.16 ± 0.01c	365.04 ± 41.89a	7.24 ± 0.26a
		2	2.46 ± 0.19ab	74.12 ± 9.76b	56.23 ± 3.62b	0.29 ± 0.02b	405.16 ± 22.44a	6.03 ± 0.38b
		3	3.110.35 ± a	39.02 ± 15.71b	36.09 ± 3.79c	0.37 ± 0.01a	516.77 ± 123.48a	4.97 ± 0.16c
	NS	1	1.37 ± 0.11c	673.46 ± 18.10a	241.97 ± 7.94a	0.15 ± 0.01c	395.63 ± 29.49b	7.30 ± 0.32a
		2	2.40 ± 0.17b	94.07 ± 11.77b	66.29 ± 4.85b	0.26 ± 0.01b	457.39 ± 34.49ab	6.38 ± 0.44a
		3	3.53 ± 0.18a	26.46 ± 2.17c	29.07 ± 2.42c	0.42 ± 0.04a	485.42 ± 16.18a	5.29 ± 0.30b
	CK	1	2.29 ± 0.05b	1003.61 ± 65.00a	361.80 ± 16.72a	0.10 ± 0.00b	244.28 ± 12.35b	9.86 ± 0.27a
		2	2.58 ± 0.07a	476.74 ± 10.71b	193.41 ± 6.09b	0.16 ± 0.01b	353.41 ± 11.34a	9.17 ± 0.15ab
		3	1.95 ± 0.10c	99.16 ± 1.98c	60.91 ± 3.94c	0.34 ± 0.04a	373.37 ± 20.20a	8.84 ± 0.16b

Table S3. Pearson correlation coefficient matrix among root and leaf traits of 15 plant species. The lower and upper triangles represent the correlation coefficient and p values, respectively. The values of the bold text indicate a significant correlation at $p < 0.05$.

	RD	SRL	SRA	RTD	RCC	RNC	DOF	LDMC	SLA	LTD	LCC	LNC
RD		**0.001**	**0.001**	**0.009**	**<0.001**	**0.002**	0.157	0.179	**0.007**	0.051	0.592	0.726
SRL	**−0.47**		**<0.001**	**<0.001**	**<0.001**	**0.001**	0.738	0.458	0.320	0.516	0.762	0.832
SRA	**−0.49**	**0.99**		**<0.001**	**<0.001**	**0.001**	0.911	0.519	0.308	0.310	0.999	0.770
RTD	**0.39**	**−0.77**	**−0.82**		**<0.001**	**0.001**	0.349	0.888	0.883	0.601	0.238	0.358
RCC	**0.50**	**−0.52**	**−0.54**	**0.58**		**<0.001**	0.316	0.854	0.490	0.420	**0.003**	0.298
RNC	**−0.46**	**0.47**	**0.48**	**−0.49**	**−0.59**		0.414	0.641	0.181	0.163	0.693	0.165
DOF	−0.22	0.05	0.02	0.14	0.15	−0.13		**<0.001**	**<0.001**	**0.030**	0.255	**0.048**
LDMC	0.20	−0.11	−0.10	−0.02	−0.03	0.07	**−0.83**		**<0.001**	**0.001**	0.873	0.156
SLA	**−0.40**	0.15	0.16	−0.02	−0.11	0.20	**0.67**	**−0.65**		**<0.001**	0.707	0.808
LTD	0.29	−0.10	−0.16	0.08	0.12	−0.21	**−0.32**	**0.50**	**−0.66**		0.314	0.577
LCC	0.08	−0.05	0.00	−0.18	**−0.44**	−0.06	−0.17	−0.03	−0.06	−0.15		0.464
LNC	−0.05	0.03	0.05	−0.14	−0.16	−0.21	**−0.30**	0.22	−0.04	0.09	0.11	
